# An alternative flow cytometry strategy for peripheral blood dendritic cell enumeration in the setting of repetitive GM-CSF dosing

**DOI:** 10.1186/1479-5876-4-18

**Published:** 2006-04-24

**Authors:** Kehui Wang, Kevin P Nishimoto, Rita S Mehta, Edward L Nelson

**Affiliations:** 1 Department of Medicine, Division of Hematology/Oncology, School of Medicine, University of California, Irvine, USA; 2 Department of Molecular Biology & Biochemistry, School of Biological Sciences, University of California, Irvine, USA; 3 Center for Immunology, University of California, Irvine, USA

## Abstract

**Background:**

Enumeration of circulating peripheral blood dendritic cells (DCs) is complicated by the absence of a unique cell surface marker expressed on all DC subsets and by the use of various biological adjuvants to modulate the DC compartment, including granulocyte macrophage colony stimulating factor (GM-CSF). Common methods employ a cocktail of antibodies, typically including anti-CD14, to define a lineage negative, MHC class II positive, putative DC population. Reported flow cytometry protocols include highly variable gating strategies and DC identification criteria. Increasing appreciation of DC pleiomorphism, GM-CSF biology, and recognition of CD14 expression in some DC subsets led us to consider an alternative lineage cocktail to improve identification of the circulating DC pool.

**Methods:**

Standard whole blood staining with appropriate fluorochrome conjugated antibodies to MHC class II and either standard CD14 containing, or an alternate CD66acde containing, lineage cocktail was performed on samples obtained from normal donors and breast cancer patients before and after administration of dose-dense, cytotoxic chemotherapy with daily GM-CSF hematopoetic growth factor support. Putative DCs were enumerated by standard flow cytometry. Data set differences were evaluated using two tailed Mann-Whitney or Wilcoxon signed rank tests. Cellular morphology was examined in cell-sorted populations from post GM-CSF samples.

**Results:**

Use of either antibody cocktail defined comparably sized lineage negative, MHC class II positive populations in normal donors and at baseline in cancer patients. However, selection of lineage negative subsets with increasing MHC class II expression levels yielded larger putative DC populations identified with the alternate cocktail. Both cocktails yielded highly reproducible data. Use of the alternate cocktail: 1) yielded a putative DC population, post GM-CSF that was more homogenous and consistent with DCs, 2) resulted in less data variation across gating strategies, and 3) resulted in more uniform and concordant longitudinal data, consistent with established GM-CSF biological activity.

**Conclusion:**

An alternative lineage negative cocktail substituting anti-CD66 antibody for anti-CD14 is a viable option for enumerating the circulating DC population, potentially more accurately defining the circulating DC pool by including CD14 positive immature DCs, and thus, may give more reliable data, particularly in the setting of sustained GM-CSF administration.

## Background

The recognition of dendritic cells (DCs) as the most potent antigen-presenting and immunostimulatory cell [1] has led to their incorporation into various immunotherapeutic and immunomodulatory strategies and has prompted the development of flow cytometry strategies for monitoring DCs. Monitoring of longitudinal changes in human DC populations necessitates evaluation of peripheral blood circulating DCs, as repeated lymph node biopsies are impractical. This ability to accurately monitor potential modulations of DCs is challenged by DC phenotypic pleiomorphism. DCs can manifest several phenotypes, including immature and mature [1-4], myeloid or type 1 (DC1) and lymphoid or type 2 (DC2). However, as there is no one marker that uniquely identifies DCs, analysis of DC populations and their modulations must be carefully interpreted.

Granulocyte macrophage colony stimulating factor (GM-CSF), a glycoprotein hematopoetic growth factor with diverse effects [5-12], Table 1, a known trophic factor for DCs, and one of the major biological adjuvants being employed to modulate DC numbers and activity, primarily targets myeloid DCs or the DC1 subset. The ability of GM-CSF to increase the bone marrow production of both granulocytes and monocytes is well documented, but it also has been consistently reported to activate various cell populations and induce MHC class II expression [13-25]. This raises concerns regarding the accuracy of flow cytometry evaluations of DCs in the peripheral blood compartment using classic lineage negative, MHC class II positive criteria, particularly in the setting of GM-CSF administration.

**Table 1:** Diverse biological activities of GM-CSF.

*In vitro *activation of macrophages, monocytes, and dendritic cells [26–30].
*In vivo *administration activates monocyte at low doses in clinical studies [31–33].
Increases antigen processing and presentation by Macrophages [34–36].
Enhanced *in vitro *tumoricidal activity of PBMC for human melanoma cells [26].
Induces macrophage production of an angiogenesis inhibitor [37, 38].

The cytometric evaluation of DCs is complicated because unlike other leukocytes, there is no single cell surface or cytoplasmic marker for all DC subsets [2,3] and there is no consensus on the most appropriate flow cytometry protocol. Although several commercially available DC-specific antibodies have been used to select or enumerate DC subsets, each identifies only a limited subset of DCs. The most widely used criteria for defining circulating DCs is lineage negative (neither lymphocytes nor monocytes nor NK cells) and MHC class II positive. The classic lineage negative antibody cocktails incorporate antibodies to T lymphocytes (anti-CD3), B lymphocytes (anti CD19 and/or anti-CD20), NK cells (anti-CD16 and/or anti-CD56) and monocytes (anti-CD14). However, low level CD14 expression by immature DCs and type 1 DC precursors (pDC1) [2] and the expression of CD16 by a subset of DCs [3,39,40] can lead to the potential incorrect assignment of cells. Additionally, various disease states, recovery from myelosuppressive chemotherapy, and/or repetitive GM-CSF administration can increase the number of circulating MHC class II positive cells complicating the use of these cocktails [14,41-47] and imparting further error to the methodology. We postulated that an antibody cocktail that would identify granulocytes, NK cells, lymphocyte lineages, and activated monocytes in whole blood analyses would potentially provide a more accurate enumeration of circulating DCs. Members of the CD66 family, recognized by commercially available monoclonal antibodies, are widely expressed on granulocytes, NK cells, lymphocytes, and activated monocytes/macrophages [48-55] and provide candidate antibodies for a lineage negative cocktail that would permit more consistent identification of the circulating DC population, even in the setting of repeated administration of the biological adjuvant, GM-CSF.

## Methods

### Blood samples

All human blood samples were collected in accordance with IRB reviewed and approved research protocols. Anonymous normal donor samples from adult subjects, 23 to 55 years of age, were obtained through the normal blood donor program administered and run by the UCI GCRC. Subjects receiving dose-dense chemotherapy for a diagnosis of breast adenocarcinoma consisting of doxorubicin (Adriamycin) 60 mg/m^2 ^d1 followed by cyclophosphamide (Cytoxan) 600 mg/m^2 ^d1, administered in a 14 day cycle received 10 days of GM-CSF at the standard hematopoetic support dose of 250 ug/m^2 ^administered by subcutaneous (SC) injection starting on day 3, under an IRB approved protocol. GM-CSF administration terminated ≥ 24 hours before the next administration of cytotoxic drugs. Samples from these subjects constitute the "patient" cohort. Standard phlebotomy was performed using EDTA containing collection tubes prior to initiation of chemotherapy, "baseline" and after the 10 days of GM-CSF.

### Whole blood staining

Two hundred microliters of well-mixed whole blood was used for each analysis. All elements of the procedure were carried out at room temperature unless otherwise noted. Antibodies were added to these samples and incubated for 60 minutes in the dark, with frequent agitation. After the addition of red cell lysis media ACK (MP Biomedical, Irvine, CA) the mixture was incubated for an additional 15 minutes. Cells were collected by centrifugation at 1000 RPM × 5 minutes, the supernatant was discarded, and the cell pellet resuspended in staining media consisting of phosphate buffered saline, pH 7.4, containing 3% Fetal Clone III (Hyclone, Logan, UT) and 0.1% sodium azide as a wash step. After this wash, the cell pellet was resuspended in 500 μl of staining media containing 1% fresh paraformaldehyde. Samples were stored at 4 C in the dark for no more than 48 hours before flow cytometry analysis.

### Flow cytometry, FACS, and antibodies

Evaluation of nucleated cells from whole blood specimens was performed by standard flow cytometry on a FACScalibur (Becton Dickinson, Franklin Lakes, NJ) instrument with identical set up parameters between samples, using appropriate primary or fluorochrome conjugated monoclonal antibodies against cell surface markers to CD3, CD14, CD20, CD56, CD66acde, CD80, CD83 CD86, appropriate isotype control, and secondary antibodies (Caltag Laboratories, Burlingame, CA), CD11c, (BD Biosciences, San Diego, CA), and MHC II (Ancell Bayport, MN) as noted in Table 2. Flow cytometry data was analyzed using FlowJo software (Tree Star, Ashland, OR).

**Table 2:** Antibodies employed in these studies

Antigen	Fluorochrome	Clone	Isotype	Vendor	Volume
CD3	FITC	S4.1	IgG2a	Caltag	5 ul
CD11c	PE/Cy5	B-ly6	IgG1	BD Biosciences	10 ul
CD14	FITC	TüK4	IgG2a	Caltag	5 ul
CD20	FITC	HI47	IgG3	Caltag	5 ul
CD56	FITC	MEM-188	IgG2a	Caltag	5 ul
CD66acde	FITC	CLB-gran/10	IgG1	Caltag	5 ul
CD80	Tricolor	MEM-233	IgG1	Caltag	5 ul
CD83	Tricolor	HB15e	IgG1	Caltag	5 ul
CD86	Tricolor	BU63	IgG1	Caltag	5 ul
MHC II	PE	TDR31.1	IgG1	Ancell	4 ul

**Figure 1:**
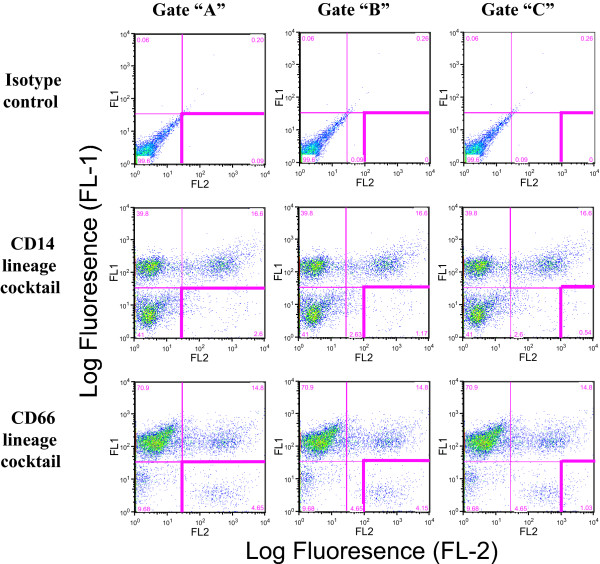
**Alternate "gates" for flow cytometric enumeration of circulating dendritic cells**. Representative dot plots of two color flow cytometric evaluations from one of the six anonymous "normal" donor samples. All plots depict log fluorescence of FL-1 (Lineage – FITC) on the "Y axis" and FL-2 on the "X" axis (MHC II – PE) with collection of a minimum of 50,000 events. The left hand column depicts "Gate A", classic quadrant gates. The middle column depicts the least restrictive of the arbitrarily set MHC class II high expression subset "Gate B". The right hand column depicts the most restrictive MHC class II high expressing population, "Gate C". The first row represents isotype control analyses and gate settings that have 0.09 %, 0.00%, 0.00 % ≥ background, respectively.

There is no consensus for the most appropriate gating strategy for circulating DC enumeration. Classic quadrant gates [56-58], more restricted MHC class II high expressing gates [59,60], and even more inclusive gates [61] have been employed for DC enumeration with and without restriction of the examined cellular subset by side scatter & forward scatter gating, complicating direct comparison of results from different groups. Thus, we evaluated three different gating profiles in two color analyses of lineage negative, MHC class II positive putative DC populations. We set standard quadrant gates from isotype control samples with less than 0.2% background in all positive gates, "Gate A". DCs generally have high-level expression of MHC class II, thus we also examined various levels of higher MHC class II expression within this gate. In our experience with whole blood staining and analyses, discrete populations with different levels of MHC class II expression are rarely observed. Thus, these gates were arbitrarily set at ≥ 10^2 ^log FL-2 fluorescence, "Gate B", or ≥ 10^3 ^log FL-2 fluorescence, "Gate C", see Figure 1. Although there are certainly other gating strategies, these three strategies represent a range of restriction for the level of MHC class II expression in the lineage negative, MHC II positive, putative DC population.

Cells for photomicrography were obtained by fluorescent activated cell sorting (FACS) collecting MHC class II positive, lineage negative and lineage positive populations using "Gate B" settings. These samples were used to generate cytospin preparations. Cytospin preparations were air dried and stained with standard Wright-Giemsa. Photomicrographs were obtained using a cooled color CCD camera (Diagnostic Instruments, Sterling Heights, MI).

### Statistical methods

The two-tailed Wilcoxon signed rank tests were used to test for significant differences between comparisons conducted within individual sample sets, e.g., normal or patient sets. The two-tailed Mann-Whitney tests were used to test for significant differences in intergroup comparisons. Pearson's R was calculated to assess the degree of correlation between replicate analyses from given samples as a measure of reproducibility in this whole blood analytical strategy. Figures were generated using Graph Pad Prism (Graph Pad Software, San Diego CA) and Microsoft Excel (Microsoft Corp., Redmond, WA) software programs with statistical analyses performed using SAS software (SAS Institute Inc., Cary NC).

## Results

### DC enumeration by CD14 and CD66 lineage cocktails in the absence of GM-CSF

It is widely believed that the proportion of the circulating leukocyte pool that constitutes the circulating dendritic cell population is a small percentage. We evaluated the effect of alternate gating strategies on the number of enumerated DCs from whole blood samples: Gate A represents the classic quadrant gate, Gate B and Gate C employ increasing restrictions on high level MHC class II expression in the lineage negative population, Figure 1. The boundaries for Gates B & C were arbitrarily set at > 10^2 ^and > 10^3 ^on the log FL-2 fluorescence scale within Gate A, respectively. The isotype control background for these gate settings were 0.09 %, 0.00%, 0.00 %, respectively. Enumeration of putative DCs, in the respective gates, yielded values of; 2.6%, 1.17%, and 0.54%, using the CD14 containing lineage cocktail and using the CD66acde containing lineage cocktail; 4.65%, 4.15%, 1.03%. The absence of discrete populations of cells with different levels of MHC class II expression and the arbitrary nature of setting these alternate gates accentuate the difficulties of comparing data between groups in the absence of detailed gating strategy descriptions.

The basis for considering alternative DC enumeration flow cytometry strategies is the potential for incorrect classification of leukocytes, either as DCs or as non-DCs, particularly in the setting of GM-CSF administration. To gain insight into the extent of this potential error, we examined several specimens with three-color, flow cytometric analysis to determine the distribution of cells expressing CD11c, CD14, CD80, CD83, or CD86, in the various populations delineated by lineage cocktail and MHC class II reactivity. Table 3 summarizes a representative set of analyses from a subject sample after receiving GM-CSF. The vast majority of the CD14 expressing population resided in the lineage positive, MHC class II positive cell subset for both lineage cocktails, 82% and 76%, respectively. Increasing restriction on high MHC class II expression led to an enrichment of the percentage of cells expressing CD14 for both cocktails, although to a greater extent in the CD66acde cocktail. This accounted for the increase in identified putative DC in the CD66acde cocktail analyses vs. the CD14 cocktail analyses. CD11c expression has been associated with DCs in the presence of MHC class II expression. Slightly less than half of the MHC class II positive, CD11c positive cells resided in the lineage positive population for samples analyzed with the CD14 lineage cocktail, while this percentage was much higher in the CD66acde lineage cocktail analysis. Increasing restriction on high MHC class II expression led to an enrichment of the percentage of cells residing within the gated population expressing either CD11c or CD14 with the CD66acde cocktail, whereas in the CD14 cocktail analyses the proportion of CD11c positive cells varied little across the three gates. Interestingly, approximately identical proportions of CD66acde lineage negative, MHC class II positive putative DCs enumerated by the CD66acde cocktail expressed CD14 or CD11c, supporting their appropriate classification as circulating DCs. Although the proportion of nucleated cells expressing CD80, CD83, or CD86 resided solely in the MHC class II positive pool, there were slightly higher proportions identified in the lineage negative, MHC class II positive populations from the CD66acde cocktail analyses. Nearly identical trends to those depicted in Table 3 were seen in samples from subjects not receiving GM-CSF. Together, these data suggest that monocytes are classified similarly by both cocktails and support the under classification of DCs by analyses using the CD14 cocktail.

**Table 3:** Expression of select markers on populations categorized by lineage cocktail reactivity and MHC class II expression. This table lists the percentage of nucleated cells residing in each designated gate for each of the two lineage (Lin) cocktails. The first data column, "Total", represents the total percentage of cells within the designated gates described in the far left hand column. Subsequent data columns denote the percentage of cells residing within the designated gate expressing the designated cell surface molecule designated in the top row. Numbers in parentheses represent the percentage molecule expressing cells in the sample; the "ungated" value represents the total percentage.

Lineage cocktail & gate	Percentage of cells in designated FL-1 Fl-2 gate (portion of ungated marker + population)					
	
	Total	CD11c +	CD14 +	CD80 +	CD83 +	CD86 +
CD14 cocktail						
Ungated		(25)	(11.4)	(3.9)	(3.4)	(5.3)
Lin – MHC II -	50.2	2.9 (1.4)	2.5 (1.3)	0 (0)	0 (0)	0 (0)
Lin + MHC II -	13.5	0.6 (0.1)	2.0 (0.3)	0 (0)	0 (0)	0 (0)
Lin + MHC II +	12.9	82.7 (10.6)	72.0 (9.3)	30.5 (3.9)	25.6 (3.3)	39.1 (5.0)
Lin – MHC II +	23.4	52.8 (12.3)	2.5 (0.6)	0.04 (0.01)	0.4 (0.1)	0.9 (0.3)
Gate "B"	6.9	67.9 (*4.7*)	4.6 (*0.3*)	0.1 (*0.01*)	0 (*0*)	0.15 (*0.01*)
Gate "C"	0.11	66.7 (*0.1*)	18.2 (*0.02*)	10 (*0.01*)	0 (*0*)	0.06 (*0*)
CD66 cocktail						
Ungated		(23.8)	(12.3)	(3.1)	(2.8)	(5.8)
Lin – MHC II-	12.9	1.7 (0.2)	0.2 (0.02)	0.08 (0.01)	0 (0)	0 (0)
Lin + MHC II -	52	3 (1.6)	1.1 (0.6)	0 (0)	0.02 (0.01)	0 (0)
Lin + MHC II +	28.7	66.8 (19.2)	32.5 (9.3)	10.4 (3.0)	9.9 (2.8)	18.3 (5.3)
Lin – MHC II +	6.4	44 (2.8)	36.9 (2.4)	1.58 (0.1)	1.25 (0.01)	8.5 (0.5)
Gate "B"	3.8	68.8 (*2.6*)	61.3 (*2.3*)	2.7 (*0.1*)	1.9 (*0.01*)	14.4 (*0.5*)
Gate "C"	1.4	95 (*1.3*)	89.9 (*1.3*)	7 (*0.1*)	5.7 (*0.01*)	27.4 (*0.4*)

**Figure 2:**
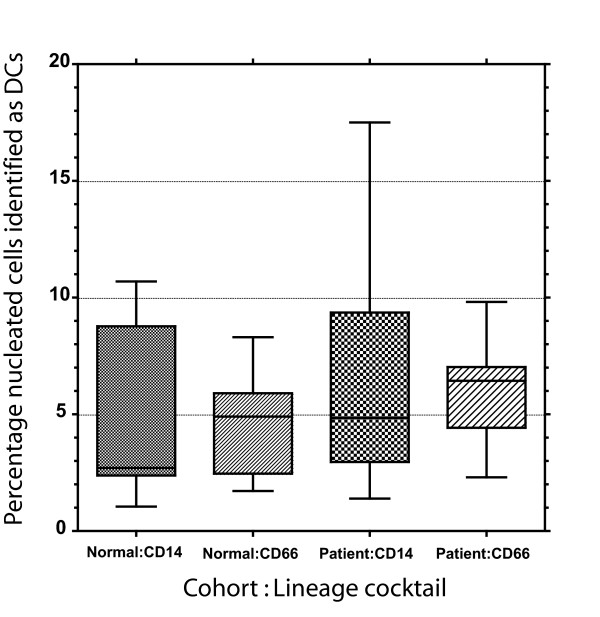
**Comparison of CD14 and CD66acde containing lineage cocktails for flow cytometry DC enumeration in the absence of repeated GM-CSF administration**. Each set of percent DC determinations for the designated samples and lineage cocktail formulation are depicted as box and whisker plots depicting the median, bold line within the box, the bounds of the 25^th ^and 75^th ^percentile, the box, and the data set range, the whisker.

Given the fact that immature DCs can have low level CD14 expression, we predicted that even in the absence of GM-CSF administration the alternate cocktail would yield a higher proportion of circulating leukocytes identified as DCs. Thus, we evaluated 18 samples from both normal donors and pre-chemotherapy baseline samples from subjects that would go on to receive GM-CSF. The DC populations were initially enumerated using the criteria of lineage negative, MHC class II positive defined by isotype control quadrant gating at ≤ 0.2 % thresholds, Gate A. There was a trend toward the CD66acde lineage cocktail identifying a larger population of putative DCs, although it did not reach statistical significance, Figure 2. The overlapping box and whisker plots from all samples suggests that these two cocktails identify comparable numbers of circulating DCs when employed in a commonly accepted gating strategy, Gate A, in the absence of the biological adjuvant GM-CSF.

### Alternate gating strategies with the CD14 lineage cocktail impart greater variability in enumerated DCs than with the CD66 lineage cocktail

**Figure 3:**
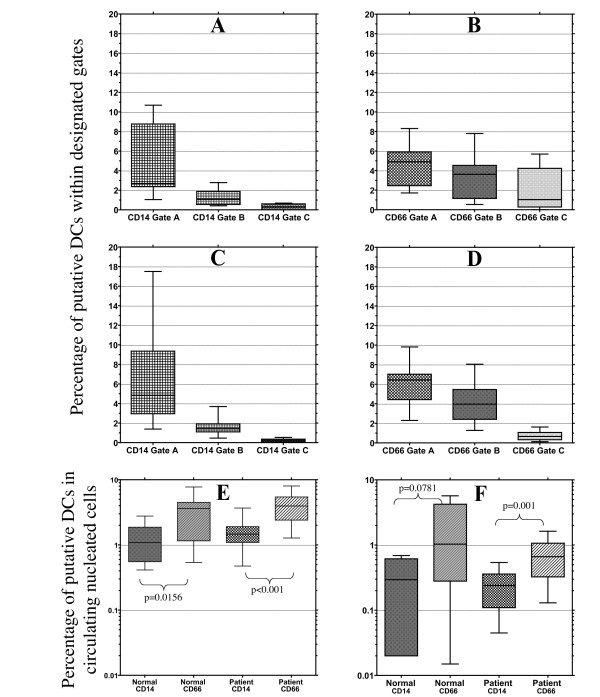
**Effect of alternate gating strategies on DC enumeration by lineage cocktail**. Box and whisker plots of compiled data for percentage DCs in normal and patient samples using the three alternate gating strategies. Normal samples evaluated using the CD14 containing lineage cocktail are depicted in Panel A and evaluated using the CD66 containing lineage cocktail depicted in Panel B, using the designated gates on the "X" axis. Patient samples evaluated using the CD14 containing lineage cocktail are depicted in Panel C and evaluated using the CD66 containing lineage cocktail depicted in Panel D, using the designated gates on the "X" axis. Within each sample set the differences between analyses using each of the three gates are statistically significant at p values ≤ 0.0312. Direct comparison of enumerated values for putative DCs in both "Normal and "Patient" baseline samples as detected using each lineage cocktail with Gate B, Panel E, and with Gate C, Panel F, reveals consistent and statistically significant differences, as noted, between determinations using the different cocktails.

The three gating strategies for each lineage cocktail were applied to the normal donor samples, Figure 3, **panels A & B**, and baseline patient samples, Figure 3, **panels C & D, **sets described above. The proportion of circulating leukocytes identified as DCs decreased with restriction to higher level MHC class II expression, Gates B & C. Within each sample, the identified DC population varied to a greater extent, relative to the employed gating strategy, using the CD14 containing lineage negative cocktail relative to the CD66 containing lineage negative cocktail. In the setting of the arbitrarily set MHC class II high gates, the number of DCs enumerated in analyses using the CD66 lineage were, as predicted, significantly higher than the population identified by identical analyses using the CD14 lineage cocktail, in the normal sample set p = 0.0156 and p = 0.0781 for Gate B, Figure 3E, and Gate C, Figure 3F, respectively and in the experimental sample set p < 0.001 and p = 0.001 for Gate B, Figure 3E, and Gate C, Figure 3F, respectively. This result is not unexpected given the low level CD14 expression on immature DCs and the prediction that a large proportion of circulating DCs are likely to have the immature phenotype.

### Longitudinal change in putative DC populations in the setting of repeated GM-CSF dosage

**Figure 4:**
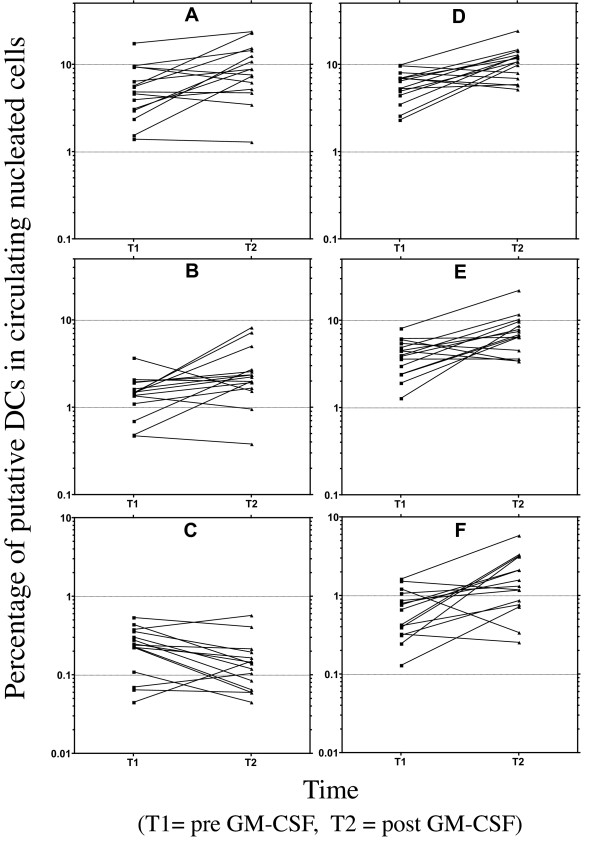
**Longitudinal changes in circulating DCs in the setting of daily GM-CSF**. Individual plots of data derived from patient samples using the various gating strategies and the two lineage cocktails. Each line depicts the longitudinal change in the percentage of nucleated cells identified as DCs in individual subject's samples at baseline (T1) and after 10 days of daily GM-CSF (T2). The left hand column represents determinations using the CD14 containing lineage cocktail and the right hand column the determinations using the CD66 containing cocktail. Panels A & D depict Gate A determinations. Panels B & E depict the Gate B gated population. Panels C & F depict the Gate C determination, with a change to log scale to accommodate the range of values.

Individuals receiving dose-dense chemotherapy supported by the growth factor GM-CSF were evaluated pre-chemotherapy and after 10 days of daily GM-CSF administration. The administration of GM-CSF was generally accompanied by a longitudinal increase in number of circulating nucleated cells identified as DCs, Figure 4. The degree of increase was variable as was the baseline proportion of DCs, though the CD66acde containing cocktail analyses generally identified a larger DC population than the CD14 containing lineage cocktail. Although with restriction to higher levels of MHC class II expression in the CD14 cocktail analyses, there was a dramatic shift in the longitudinal profile; from all but three individuals having an increase in circulating DCs after GM-CSF administration in Gate A, to only three having an increase identified using Gate C, Figure 4, **panel C**. This inconsistency was not seen across the three gates for analyses using the CD66acde lineage cocktail. It is reassuring that in general, longitudinal changes were concordant between comparable analyses using the different cocktails. Given the wide range of GM-CSF effects and the widely held belief that circulating DCs represent a small percentage of circulating nucleated cells, the substantial populations of putative DCs identified by employing Gate A, > 10% of circulating nucleated cells in some individuals, seem likely to be an over estimation of the DC population. It is difficult to reconcile the near global longitudinal decrease in circulating DCs using the CD14 lineage cocktail and Gate C, Figure 4, **panel C**, with the biological activity of GM-CSF and the general agreement between the other analyses, Figure 4, **panels A, B, D, E, & F. **The discordance between longitudinal analyses using the CD14 and CD66acde lineage cocktails, accentuate the difficulties inherent in these analyses in the setting of repetitive GM-CSF administration.

### Reproducibility of DC enumeration

**Figure 5:**
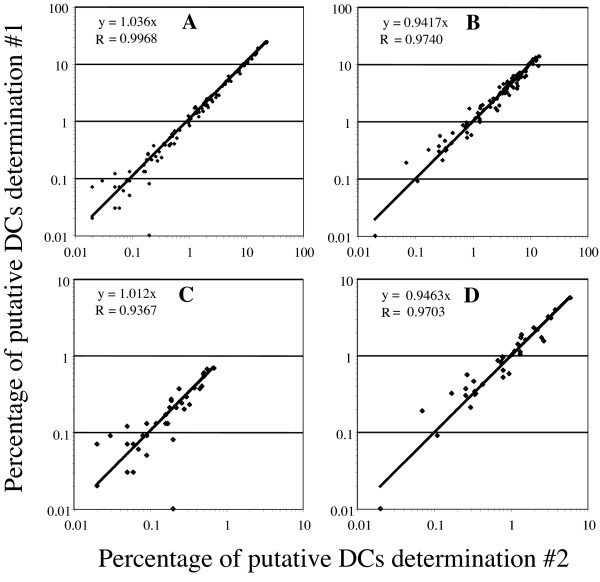
**Reproducibility of whole blood flow cytometric enumeration of DCs**. Scatter plots of independent duplicate analyses of each individual fresh whole blood sample are presented for the CD14 containing lineage cocktail, Panel A, and the CD66acde containing lineage cocktail, Panel B. There is a high degree of concordance between replicate analyses including data from all gating strategies, Panel A, (CD14 containing cocktail) with the best fit line constrained by passing through the origin and Pearson's R being y = 1.036x R = 0.9968. Panel B depicts the identical data for the CD66acde containing cocktail, y = 0.9417x R = 0.9740. Data restricted to Gate C yields a similar degree of concordance between replicate analyses with the best fit line constrained by passing through the origin and Pearson's R being y = 1.012x R = 0.9367 for CD14 lineage cocktail analyses, Panel C, and for analyses using the CD66acde containing cocktail being y = 0.9463x R = 0.9703, Panel D.

As noted above, all whole blood samples were evaluated prior to PBMC isolation and/or cryopreservation. Thus, to assess reproducibility of determinations using either lineage cocktail, replicate aliquots from individual samples were stained and analyzed. Scatter plots of these replicate determinations are depicted in Figure 5. Data derived from CD14 containing lineage cocktail determinations employing all three gates, Figure 5, **panel A**, and from CD66acde containing lineage cocktail determinations, Figure 5, **panel B**, reveal a very high level of concordance with the best fit lines as follows: y = 1.036x (Pearson's R = 0.9968) and y = 0.9417x (Pearson's R = 0.9740), respectively. Restricting the data set to Gate C, the most stringent criteria for identifying putative DCs, results in comparable levels of concordance for the analyses using the CD14 lineage cocktail, y = 1.012x (Pearson's R = 0.9367), Figure 5, **panel C**, and for the analyses using the CD66acde lineage cocktail, y = 0.9463x (Pearson's R = 0.9703), Figure 5, **panel D**. These data support the comparable reproducibility of analyses using either of these two lineage cocktails.

### The CD66 lineage cocktail identifies a more homogenous population in the setting of repeated GM-CSF administration

**Figure 6:**
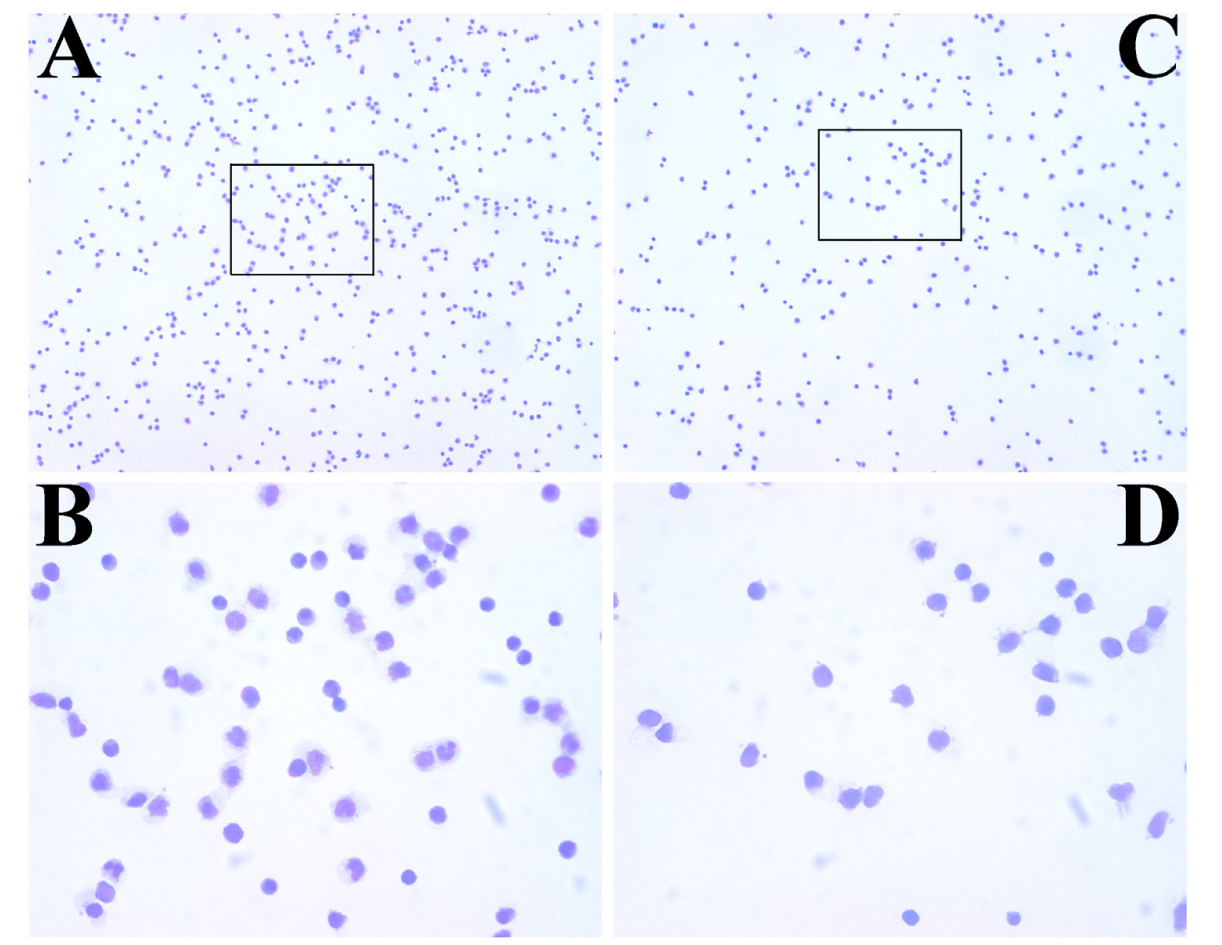
**Photomicrographs of FACS isolated populations**. Individual panels depict representative Wright-Geimsa stained cytospin preparations of MHC class II positive, lineage negative or lineage positive populations isolated by FACS using Gate B parameters from samples collected from an individual after ten consecutive days of GM-CSF following cytotoxic chemotherapy. Panels A & C depict photomicrographs (original magnification 100x) of lineage negative, MHC class II positive populations for CD14 and CD66acde containing cocktails, respectively. Panels B & D depict higher magnification (original magnification 400x) of the boxed regions in Panel A & C, respectively. These data are representative of analyses performed on three separate samples.

MHC class II positive, lineage negative and lineage positive populations obtained by standard fluorescent activated cell sorting (FACS) using Gate B were used for generating cytospin preparations from three post-GM-CSF patient samples. The limited numbers of cells present in the most restricted gate, Gate C, precluded collection and morphologic analysis of this population from these whole blood stained specimens, even in the setting of repeated GM-CSF administration. Representative photomicrographs from cytospins obtained from one sample are shown in Figure 6. Lineage negative, MHC class II positive populations isolated using the CD14 containing cocktail, Figure 6, **panels A & B**, and using the CD66acde containing cocktail, Figure 6, **panels C & D**, reveal that the lineage negative, MHC class II positive, putative DC populations isolated with the CD14 cocktail constitutes a pleiomorphic population including granulocytes and immature myeloid cells, best seen at higher power (400 × original magnification) Figure 6, **panel B**. In contrast, the CD66acde containing lineage negative, MHC class II positive population is more homogenous and has cells with the morphology of immature DCs, Figure 6, **panel D**.

## Discussion

Various strategies to modulate elements of the DC compartment are being developed and tested. Rigorous methods for evaluating the impact of these strategies on the DC compartment are critical for efficient development and evaluation of individual strategies and for gaining mechanistic understandings of various immunomodulatory strategies. Methods for enumerating DCs should take into account our evolving understanding of the complexity of the DC compartment and the biology of putative immunomodulatory biological adjuvants.

The optimal flow cytometry strategy for enumerating DCs is not known. This is a direct consequence of the absence of a single defining cell surface marker and the phenotypic pleiomorphism of the DC compartment. All of the published and commercially available strategies have potential drawbacks, Table 4; however, different methods may be more applicable in some situations than others. Examination and careful characterization of DC subsets by multi-channel flow cytometry will facilitate increased understanding of the complicated biology of DCs. The alternative cocktail described herein is as compatible as the existing lineage cocktails, with such DC1 subset evaluations.

**Table 4:** 

***Antibody cocktails***	**Complicating factors & potential drawbacks**
Lin1 (BD Biosciences^®^) *CD3*, *CD14*, *CD16*, *CD19*, *CD20*, *CD56 negative*: *MHC class II positive*.	Low-level expression of CD14 by "immature" DCs or pDC1 [2, 3] Expression of CD16 by a subset of DCs [3, 39, 40]
*CD14*, *CD16 negative*: *MHC class II*, *CD33 positive *[56, 57]	Low-level expression of CD14 by "immature" DCs or pDC1 [2, 3] Expression of CD16 by a subset of DCs [3, 39, 40] Expression pattern of CD33 [62]
***Single Antibodies***	
BDCA1, BDCA3 (Miltenyi Biotech^®^)	Identifies a limited subsets of myeloid DCs, CD1c positive subset (BDCA1) or CD141 expressing subset (BDCA3) [3, 63]
CMRF clones [3, 64]	Identify limited subsets of circulating DCs [3, 64]

In pursuing our objective of developing an alternative strategy to provide enumeration of the broader circulating myeloid DC pool than reported lineage cocktails that would be applicable to whole blood flow cytometry analysis and retain the ability to evaluate functional capacity, we investigated several potential substitute cell surface antigens that are not expressed on monocytes. CD66 proved to be the most attractive candidate marker due to its expression on granulocytes, NK cells, lymphocytes, and macrophages and absence of reported expression on DCs [48-55]. The report of reactivity in macrophages and macrophage-like myelomonocytic cell lines raised concerns for as yet unrecognized expression on myeloid DCs. Our data and recent reports that DCs do not express CD66 [65,66], however, do not justify this concern. The low level expression of CD66 family members on the more immature compartments of myelocyte development could complicate the use of this alternative cocktail in the evaluation of bone marrow or enriched progenitor cell preparations. We evaluated the two commercially available antibodies; anti-CD66acde, clone CLB-gran/10 (Caltag) and anti-CD66abce, clone Kat4c (Dako) and found both to yield similar if not identical results (data not shown).

Our analyses using both the standard CD14 and the CD66 containing lineage cocktails to enumerate DCs in normal donors and cancer patients prior to receiving cytotoxic chemotherapy and GM-CSF reveal a slightly higher DC percentage of circulating DCs in nucleated leukocytes than has generally been reported, particularly in Gates A and B. Our data is comparable to reports evaluating DC populations in cord blood. The arbitrary restriction to a high MHC class II expressing population brings our results more in line with preceding reports. Although use of the CD14 lineage cocktail sporadically yielded a suggestion of a discrete population with higher MHC class II expression, such as in Figure 1, careful examination failed to convincingly demonstrate a discrete population. We are concerned that setting arbitrary MHC class II high expression gates imparts a significant potential for bias, diminished reproducibility, and accuracy. Interestingly, recent studies report comparable percentages of circulating DCs [67,68] to those seen with the CD66 alternative cocktail employing Gate A or B. We are reassured by the reproducibility of determinations using both cocktails that is entirely comparable to similar strategies using various cocktails [32,56-58,60,61,69-73], even though some of these studies examined only the "mature", i.e. CD83 positive, circulating DC populations [32] or specific DC subsets [32,56,57,60,61,69-73]. A similar degree of inter-patient variability in longitudinal changes of putative circulating DCs was reported in the study of repetitive daily, x 7d, GM-CSF and concomitant IL-4 administration [60] and in the study of repetitive daily, x 14d, GM-CSF administration [32]. Both lineage cocktails may incorrectly classify activated immature myeloid elements, potentially myeloid suppressor cells [74-76], as putative DCs. It was somewhat surprising that the CD66 containing cocktail yielded group data with less variability across gating strategies and with greater longitudinal concordance, in the setting of daily GM-CSF administration, than the CD14 containing cocktail. Under normal circumstances CD66 and CD14 are not necessarily co-expressed on human leukocytes [77] however there is evidence for CD66 expression on activated monocytes and macrophages [54,77] suggesting that at least a proportion of CD14 cells also express CD66. Together with our limited data using three-color flow cytometry analyses of CD14 expression on lineage positive or lineage negative, MHC class II positive populations, suggest that activated monocytes or macrophages are not being routinely classified as DCs in the CD66acde cocktail analyses. It is likely that the error imparted by excluding CD14 expressing immature DCs with the standard cocktail is at least as large as any error due to inclusion of CD14 positive monocytes in the putative DC population using the CD66acde cocktail. This is supported by the observed cellular morphology of the lineage negative, MHC class II positive population from post GM-CSF samples that is more uniform and, more importantly, representative of DCs and DC precursors as previously reported [56,78] when the CD66 containing lineage cocktail is employed.

## Conclusion

We have demonstrated that substituting an antibody for CD66acde for an antibody recognizing CD14 within a cocktail of antibodies to define lineage negative, MHC class II positive populations, i.e. putative circulating DC populations, yields population sizes of comparable magnitude across different gating strategies in baseline samples from normal donors and cancer patients prior to initiation of cytotoxic chemotherapy and hematopoetic growth factor support. The data derived from use of the alternate CD66 containing cocktail is less subject to changes in gating strategies. This alternate lineage cocktail likely classifies CD14 low, MHC class II positive circulating cells, correctly as putative DCs while classifying the large majority of CD14 positive cells in the lineage positive, non-DC, population. In patients receiving cytotoxic chemotherapy and hematopoetic support with daily GM-CSF the longitudinal data obtained with the CD66 containing cocktail is more uniform and concordant across gating strategies than that obtained with the CD14 containing lineage cocktail. Finally, in representative FACS isolated lineage negative, MHC class II positive populations from such patients the putative DC population is more homogenous and representative of DCs. Together, these data support the use of this alternative lineage negative cocktail, particularly in the setting of sustained hematopoetic growth factor, e.g. GM-CSF, use.

## Competing interests

The author(s) declare that they have no competing interests.

## Authors' contributions

KW and KPN performed the analyses and participated in manuscript preparation. RSM is the principle investigator for the clinical trials and as such was primarily responsible for the conception and conduct of the clinical trial that was the source of the patient samples. ELN conceived the study, participated in the design of the clinical trial, and was responsible for conduct of these studies along with preparation of the manuscript. All authors read and approved the manuscript.
